# Preparation and Road Performance Study of Rubber–Diatomite Composite-Modified Asphalt Mixture

**DOI:** 10.3390/ma16237359

**Published:** 2023-11-26

**Authors:** Bo Tan, Youliang Su, Yuzhu Fan, Wanzhen Zhang, Qing Li

**Affiliations:** 1 College of Civil and Architecture Engineering, Guilin University of Technology, Guilin 541004, China; bbzs2004@163.com (B.T.); fxx202310@163.com (Y.F.); zhangwanzhen0818@163.com (W.Z.); liqing@glut.edu.cn (Q.L.); 2 Collaborative Innovation Center for Exploration of Nonferrous Metal Deposits and Efficient Utilization of Resources, Guilin University of Technology, Guilin 541004, China

**Keywords:** rubber, diatomite, modified asphalt, microscopic mechanism, road performance

## Abstract

To examine the effect mechanism of rubber and diatomite on asphalt as well as the performance of asphalt mixtures for road applications, various composite-modified asphalts are prepared using rubber and diatomite. The performance of modified asphalts with various proportions is analyzed, and the optimal dosage ratio of modifiers is determined via the response surface approach. The microstructure of rubber–diatomite composite-modified asphalt is methodically examined using Fourier transform infrared spectroscopy and scanning electron microscopy. The road performance, aging resistance, and long-term stability of asphalt mixtures are evaluated through Marshall tests, wheel tracking tests, aging wheel tracking tests, freeze–thaw splitting tests, and cyclic freeze–thaw drying aging splitting tests. The obtained results reveal that asphalt with 22% rubber and 4% diatomite exhibits the best overall performance. The composite-modified asphalt essentially demonstrates the physical blending between rubber powder, diatomite, and base asphalt. The asphalt built from them formed a uniform and stable overall structure. Compared with rubber asphalt and rubber–SBS composite-modified asphalt, rubber–diatomite composite-modified asphalt exhibits superior road performance, including better aging resistance and long-term water stability in asphalt mixtures. This study can promote the further extensive application of rubber–diatomite-modified asphalt in road engineering, while providing new ideas for cost-saving and environmentally friendly asphalt modification.

## 1. Introduction

With the introduction of “Dual Carbon” targets, the modern theme in highway construction is shifting towards sustainability and environmental compatibility, opening new avenues of development for engineering [1,2,3]. “Dual Carbon” seeks to achieve carbon resource recycling by capturing and reusing carbon emissions, thereby reducing reliance on fossil fuels and reducing greenhouse gas emissions. As a result, road researchers are continuously exploring innovative asphalt modification techniques and environmentally friendly materials [4,5]. Simultaneously, discarded tires have emerged as one of the largest producers of solid waste globally, and this poses severe environmental challenges [6]. Recycling waste rubber powder as pavement material can solve the solid pollution of discarded tires. To realize the “dual-carbon” cycle, green environmental protection policy proposes new solutions [7,8].

Rubber is a common asphalt modifier known for its low cost, excellent high-temperature strength, and low-temperature cracking resistance [9]. Currently, composite modification techniques for rubber-modified asphalt have attracted considerable attention [10,11,12,13]. To further enhance the performance of rubber-modified asphalt, researchers have made significant efforts. For instance, De Albornoz [14] and Qian [15] investigated the synergistic effects of CR and SBS and showed that the aging and mechanical performance of asphalt mixtures produced with rubber-modified asphalt was very similar to that of asphalt mixtures based on traditional SBS-modified asphalt. Li [16] found that oxidized graphene and rubber powder, as composite materials, improved the rheological properties of asphalt at low temperatures. In addition, with reduced rubber particle size, rubber-modified asphalt has been proven to exhibit better low-temperature performance, elasticity, and adhesion [17,18]. It can be observed that there has been some progress in research on rubber composite-modified asphalt. However, rubber-modified asphalt still faces problems regarding segregation and the adverse effects of rubber particles on asphalt mixture performance [19,20,21].

In contrast, diatomite, as an excellent inorganic asphalt modifier, possesses stable chemical properties, high porosity, large surface area, strong adsorption capacity, and high melting point [22,23]. Adding diatomite to asphalt has the potential to enhance its performance at high temperatures, long-term aging resistance, and storage stability [24,25,26,27,28]. In recent years, significant research has been conducted on diatomite-modified asphalt. For example, Yang and Tan [29,30] found that the addition of diatomite to asphalt mixtures can enhance the high-temperature stability and moisture sensitivity of the materials. Liang et al. [31] found that rubber particles and diatomite are able to enhance the high-temperature, low-temperature, and viscoelastic properties of asphalt mixtures. Some investigations have also suggested that the combined use of diatomite and other modifiers can increase the road performance of asphalt mixtures [24,32]. 

From the literature mentioned above, it is clear that rubber powder offers advantages in terms of high temperature resistance and low-temperature crack resistance, whereas diatomite is able to improve temperature stability, moisture sensitivity, and resistance to increase the aging of asphalt. Previous studies in the literature have not reported on the effects of combined use of rubber and diatomite in composite-modified asphalt, and research on the micro-mechanisms, aging performance, and long-term water stability of rubber–diatomite composite-modified asphalt is still limited. If composite-modified asphalt is able to combine the advantages of diatomite and crumb rubber-modified asphalt, it may result in a more desirable and environmentally friendly pavement material [33].

The present investigation aims to improve the mechanical behavior of the base asphalt by adding rubber powder and diatomite, using the Design Expert surface response method [34,35] to investigate the optimal mixing ratios for these two modifiers and analyze their effect on the performance. Scanning electron microscopy and infrared spectroscopy are also employed to explore the microstructure and modification mechanisms of rubber–diatomite composite-modified asphalt. Various tests such as the Marshall test, rutting test, aging rutting test, freeze–thaw splitting test, and cyclic freeze–thaw aging splitting test are utilized to comprehensively evaluate the road performance of rubber–diatomite composite-modified asphalt mixtures. This study can promote the further extensive application of rubber–diatomite-modified asphalt in road engineering, while providing new ideas for cost-saving and environmentally friendly asphalt modification.

## 2. Materials and Methods

### 2.1. Raw Materials

Asphalt base: 70# base asphalt base produced by Guangxi Jiaoke Group New Materials Technology Co., Ltd. in Nanning, China, where the corresponding basic technical specifications are provided in Table 1. Its relevant parameters have been obtained based on the standard test methods for bitumen and bituminous mixtures for road construction (JTG E20-2011) [36].

Rubber powder: Rubber powder produced by recycling waste tires from Guangxi Jiaoke Group New Materials Technology Co., Ltd. in China is selected. The rubber powder used for asphalt processing is (30–80) mesh rubber powder, and the corresponding physical and chemical properties are presented in Table 2. Its relevant parameters have been obtained based on the standard test methods for bitumen and bituminous mixtures for road construction (JTG E20-2011) [36].

Diatomite: Diatomite is obtained by self-purification using ordinary diatomite with a density of 2.15 g/cm^3^ and an average particle size of 15.22 µm. The oil absorption rate is 1.45 g/cm^3^, the specific surface area is 47.65 g/m^2^, the pore volume is 0.02 g/cm^3^, and the average pore radius is 2.12 nm. The corresponding basic technical specifications are given in Table 3.

### 2.2. Mixture Aggregates

The coarse aggregate of the mixture is made of crushed limestone from Guilin, Guangxi, and the fine aggregate is made of machine-made sand. The various indicators of the aggregate are tested, and the obtained results are presented in Table 4, meeting the specification requirements.

### 2.3. Mineral Fillers

The mineral powder used in the asphalt mixture test is produced and transported by a certain mineral powder factory in Guilin. The laboratory measured various technical indicators of the transported mineral powder according to the “Test Specification for Aggregates in Highway Engineering” (JTG E42-2005) [37]. The test results are presented in Table 5.

### 2.4. Experimental Design

According to the relevant experimental investigations at home and abroad, this study aimed to employ the wet method to prepare rubber–diatomite composite-modified asphalt, with the content of rubber powder at 16%, 20%, and 24% and diatomaceous earth at 4%, 8%, and 12% [38], respectively. The Design Expert approach was utilized to design the experiment, and the specific experimental design is provided in Table 6.

### 2.5. Preparation of Composite-Modified Asphalt

At present, rubber composite-modified asphalt in China mainly uses the wet method, which generally goes through four stages: raw material heating, feeding and mixing, shear dispersion, and development and storage. Using base asphalt as raw material, a certain proportion of the modifier is added after heating and stirring. The modifier is appropriately cut and ground using a high-speed cutting machine to completely grind, disperse, and swell with the base asphalt, so that the modifier can be evenly mixed with the asphalt. Finally, the composite-modified asphalt is heated and swelled to mature.

**Figure 1:**
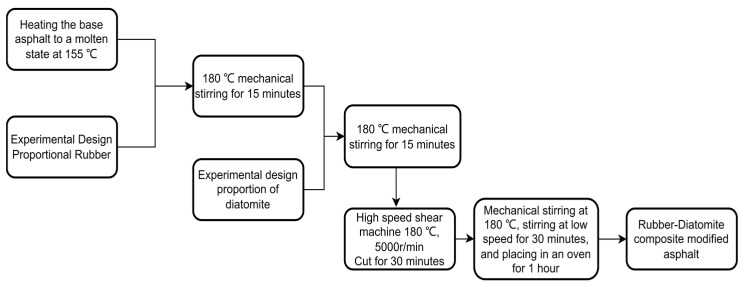
Preparation process of rubber–diatomite composite-modified asphalt.

Figure 1 shows the preparation process of rubber–diatomite composite-modified asphalt, as follows:

① Place the base asphalt in a 155 °C oven for heating and preparation. Quickly heat to 180 °C, mix the asphalt and rubber at the test ratio set for installation, and mix for 15 min with a high-speed mixer at 1000 rpm.

② Add diatomite at the experimental setting ratio to the mixed rubber-modified asphalt, and mix for 15 min with a high-speed mixer at 1000 rpm.

③ After mixing the two modifiers, maintain the temperature of the asphalt at 180 ± 5 °C. Apply 5000 rpm high-speed shearing for 30 min.

④ After cutting the asphalt, place it in a container at a constant temperature of 180 °C and mix it at high speed for 30 min. The mixed composite-modified asphalt is placed in a 160 °C oven to swell and develop for 1 h.

Finally, the rubber–diatomite-modified asphalt used in the experiment can be obtained. Afterwards, the pouring of the test specimens will be carried out in accordance with the “Test Specification for Asphalt and Asphalt Mixtures in Highway Engineering” JTGE20-2011.

### 2.6. Design of Asphalt Mixture Grading

According to the requirements of JTGF40-2004 [39] and DB45/T 1098-2014 [40], and combined with the actual situation of this study, the ARAC-13 structural layer asphalt mixture from Guangxi, China was adopted for grading. The specific mineral aggregate grading is presented in Table 7.

### 2.7. Test Process and Methods

**Figure 2:**
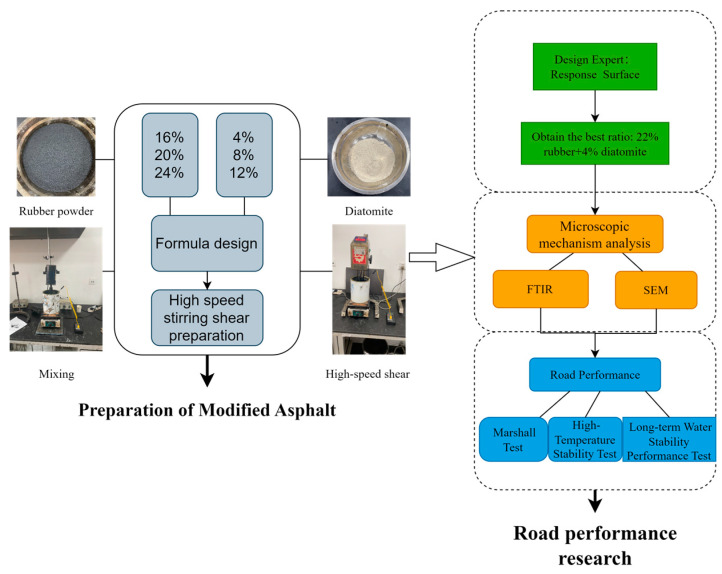
Experimental test plan.

The basic performance of diatomaceous earth composite-modified asphalt was investigated based on experiments. The three key indicators of modified asphalt (i.e., penetration, softening point, and ductility) and Brinell viscosity at 180 °C were tested according to the standard “Standard Test Methods for Bitumen and Bituminous Mixtures for Highway Engineering” (JTG E20-2011) [36]. Finally, the interaction effect of rubber and diatomite on asphalt performance was analyzed using Design Expert’s surface response graph and the optimal dosage was confirmed. The modification mechanism of the optimized modified asphalt was analyzed using infrared spectroscopy (FTIR) and scanning electron microscopy (SEM). The road performance was then verified using the Marshall test, wheel tracking test, aging wheel tracking test, cyclic freeze–thaw splitting test, and cyclic freeze–thaw drying aging splitting test applied to the asphalt mixture. The process is illustrated in Figure 2.

## 3. Asphalt Test Results and Discussion

### 3.1. Determination of the Optimal Dosage of Modifier for Composite-Modified Asphalt

#### 3.1.1. Determination of Modifier Asphalt Dosage

The test results of rubber–diatomite composite-modified asphalt are given in Table 8. 

**Figure 3:**
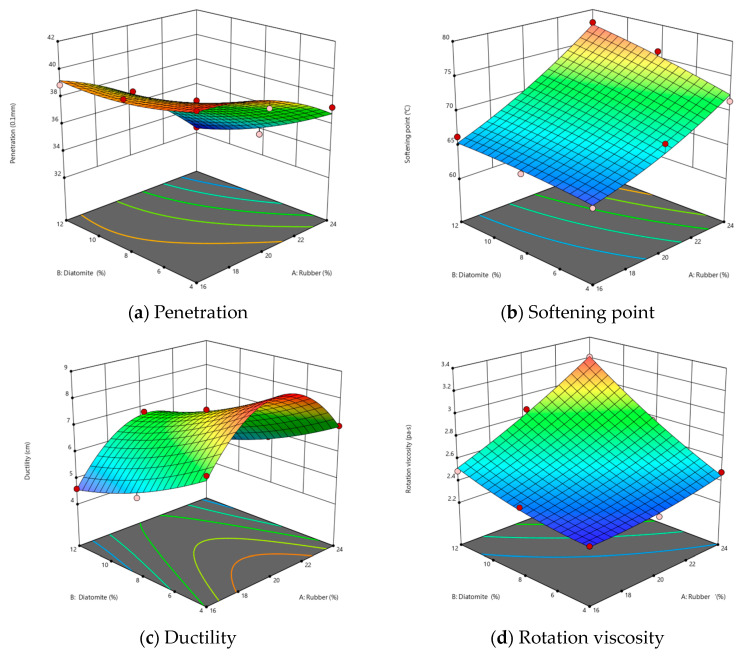
Effect of rubber/diatomite on the asphalt performance.

In order to examine the interactions between rubber and diatomite in composite-modified asphalt and to search for optimal mixing ratios for improved modification, we performed an analysis of the effects of rubber powder and diatomite on its performance via Design Expert. Figure 3 illustrates the relationship between the performance indices of composite-modified asphalt and the ratio of different composite materials, which can be described by Equations (1)–(4) denoted by *Y*_1_ to *Y*_4_ (the equation is obtained through a linear regression equation). Here, *X*_1_ and *X*_2_ represent the quantities of rubber and diatomite, respectively, while *Y*_1_, *Y*_2_, *Y*_3_, and *Y*_4_ correspond to penetration, softening point, ductility, and viscosity.

The color gradient in the figure, from blue to red, represents the gradual increase in response values. The penetration shows a negative correlation with the amounts of rubber and diatomite, and diatomite has a more obvious effect. On the contrary, the softening point shows a positive correlation with the values of rubber and diatomite, and rubber is more significant. The ductility is synergistically affected by rubber and diatomite. The viscosity possesses a positive correlation with the amounts of rubber and diatomite, and both factors have a similar effect. This suggests that the amounts of rubber and diatomite do not exhibit a simple linear relationship with the asphalt performance. Instead, there is a synergistic interaction among the three components.

(1) $$Y_{1}=37.49-2.53X_{1}-1.33X_{2}-0.8775X_{1}X_{2}-0.8633X_{1}^{2}+0.5067X_{2}^{2}$$

(2) $$Y_{2}=69.32+5.12X_{1}+1.58X_{2}+1.02X_{1}X_{2}+0.9167X_{1}^{2}-0.2833X_{2}^{2}$$

(3) $$Y_{3}=7.49+0.1667X_{1}i-1.03X_{2}+0.05X_{1}X_{2}-1.93X_{1}^{2}+0.2667X_{2}^{2}$$

(4) $$Y_{4}=2.54+0.2117X_{1}+0.2450X_{2}+0.1375X_{1}X_{2}+0.035X_{1}^{2}+0.045X_{2}^{2}$$

#### 3.1.2. Determination of Optimal Composition for Composite-Modified Asphalt

From the perspective of optimizing, considering the performance of composite-modified asphalt and the increase in rubber powder content and decrease in diatomite content, Equations (1)–(4) were integrated into the Design Expert model. The main goal was to maximize the softening point and ductility values, and lower the penetration value, while obtaining an average viscosity value. This approach aimed to determine the optimal formulation and predict the performance of the modified asphalt composite, which was subsequently verified through experiments, as presented in Table 9.

As shown in Table 9, the deviation in various indicators is minimal, and the predicted values of the equations agree with the experimental results, which indicates a high level of reliability. Finally, it was found that the optimal composition consists of 22% rubber powder and 4% diatomite.

### 3.2. Microscopic Mechanism Analysis

#### 3.2.1. Fourier Transform Infrared Spectroscopy (FTIR) Experimental Analysis

**Figure 4:**
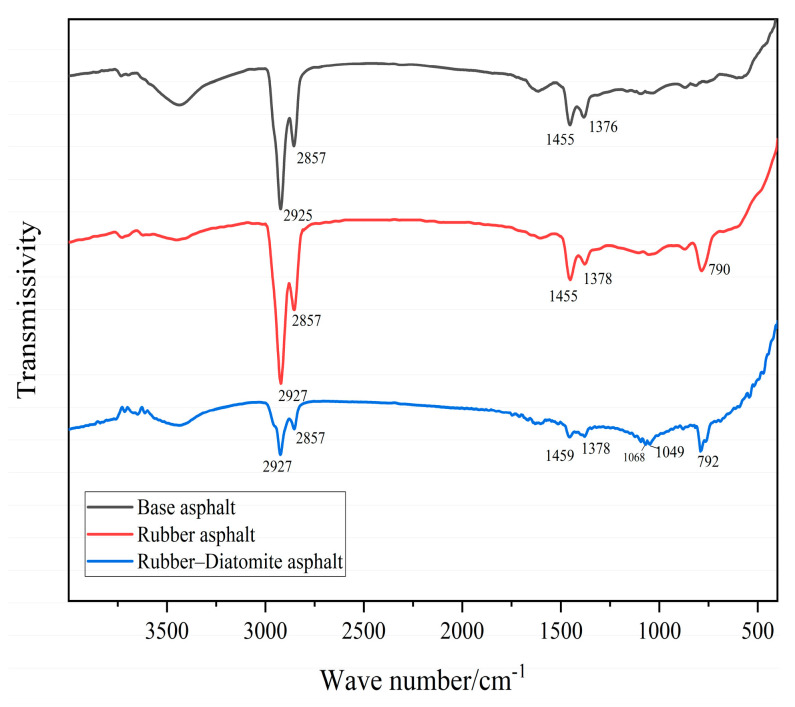
Fourier transform infrared spectroscopy.

Infrared spectroscopic analysis was conducted to investigate the base asphalt, rubber asphalt, and rubber–diatomite composite-modified asphalt, and the test results are presented in Figure 4. A slight difference is detectable around 790 cm^−1^ and 784 cm^−1^.

When comparing the infrared spectroscopy of rubber asphalt and rubber–diatomite asphalt, the only difference is in the appearance of absorption peaks at 1049 cm^−1^ and 1068 cm^−1^, which are associated with stretching and bending vibrations of the Si-O bonds. The peaks at 1049 cm^−1^ and 1068 cm^−1^ represent characteristics of Si-O stretching vibrations in diatomaceous earth, which is the main component of SiO_2_. No other variations or new absorption peaks were observed [41]. In conclusion, infrared spectroscopic comparisons of base asphalt, rubber asphalt, and rubber–diatomite samples reveal that no substantial chemical reactions ensue between rubber, diatomite, and asphalt. Rather, it appears to be a process of physical blending.

#### 3.2.2. SEM Analysis of Rubber–Diatomite

**Figure 5:**
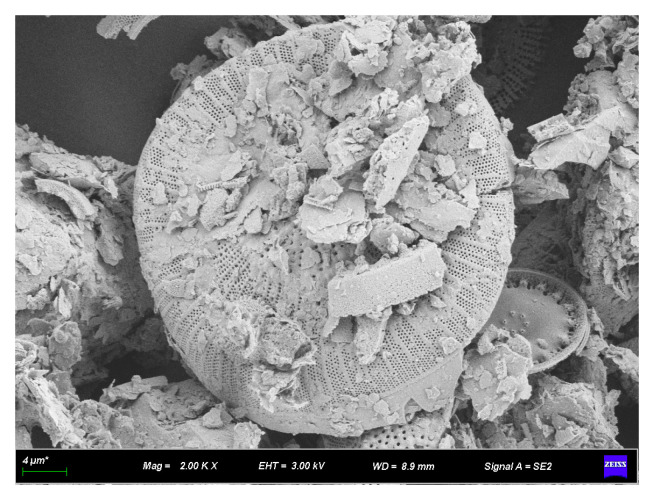
SEM appearance of diatomite (2000×).

**Figure 6:**
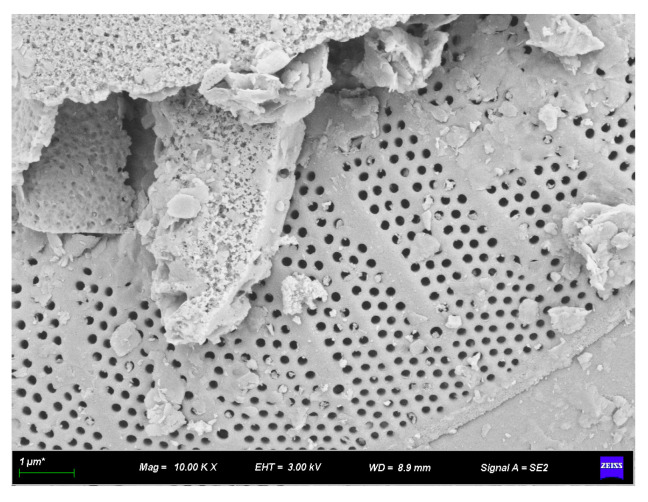
SEM appearance of diatomite (10,000×).

Figure 5 and Figure 6 illustrate the SEM images of diatomite, and Figure 6 is an enlarged view of a portion of Figure 5. These images show the commendable performance of diatomite in the framework of mineral processing and purification. The individual diatomite surface exhibits a clear, smooth, and regular contour. This feature helps to improve its surface bonding with asphalt during the modification process and facilitates the formation of a stable system between diatomite and asphalt. This, in turn, increases the performance of the modified asphalt and enhances the effectiveness of the modification.

In addition, the abundance of small pores on the surface of diatomaceous earth contributes to increasing the surface area and enhancing adsorption capacity. As a result, the asphalt is able to easily wet and coat the diatomite, which in turn increases the efficiency of diatomite modification with asphalt.

**Figure 7:**
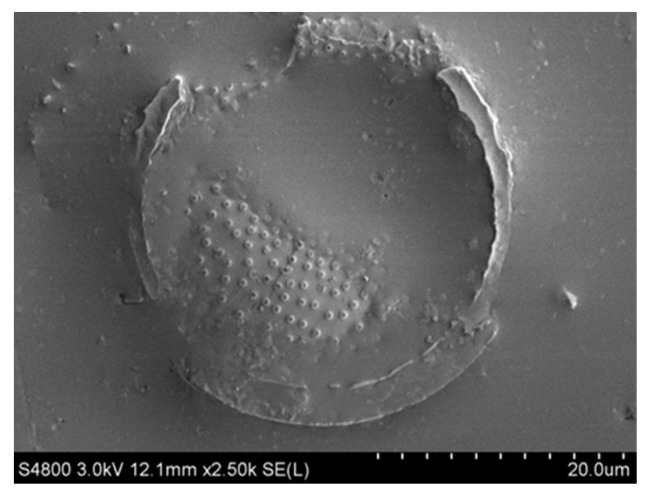
SEM image of diatomite with asphalt (2500×).

**Figure 8:**
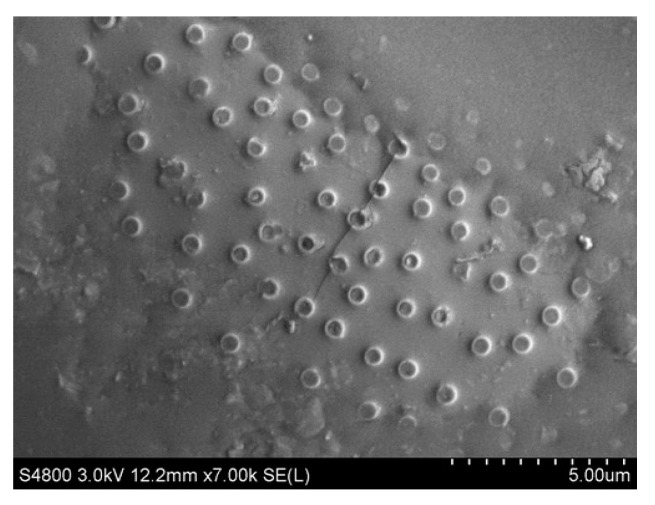
SEM image of diatomite with asphalt (7000×).

Figure 7 and Figure 8 illustrate the appearance of the bond between diatomite and asphalt, and Figure 8 is an enlarged view of a portion of Figure 7. These images display the asphalt texture on the diatomite surface, indicating that the modification of asphaltic diatomite mainly occurs through physical adsorption. The internal pores of diatomite possess a strong absorption capability and the modification process is carried out at high temperatures. At high temperatures, asphalt easily wets the diatomite surface, thereby growing the adsorption capacity and surface bonding. This indicates that diatomite has a greater tendency to absorb easily soluble components within the asphalt. During the modification process, saturated fractions and aromatic fractions are more readily drawn into the internal and surrounding pores of diatomite.

**Figure 9:**
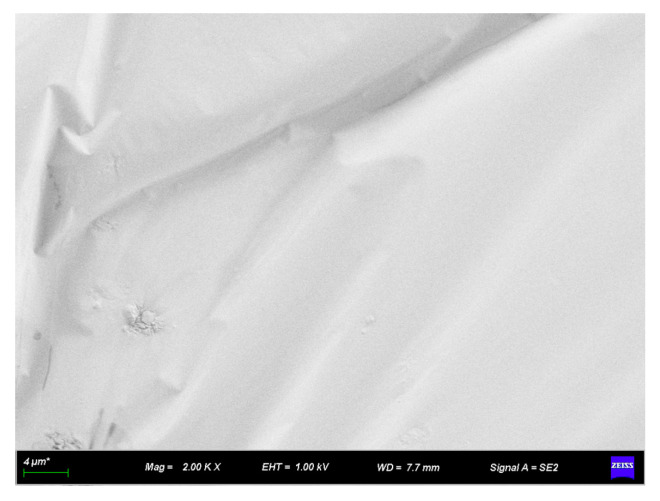
SEM appearance of base asphalt.

**Figure 10:**
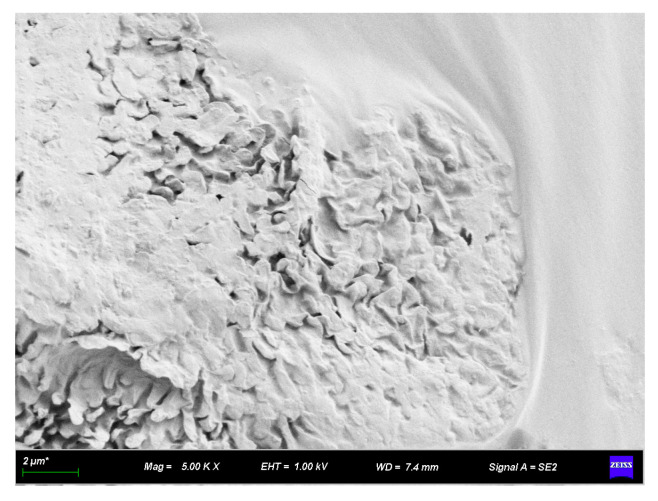
SEM appearance of rubber asphalt.

**Figure 11:**
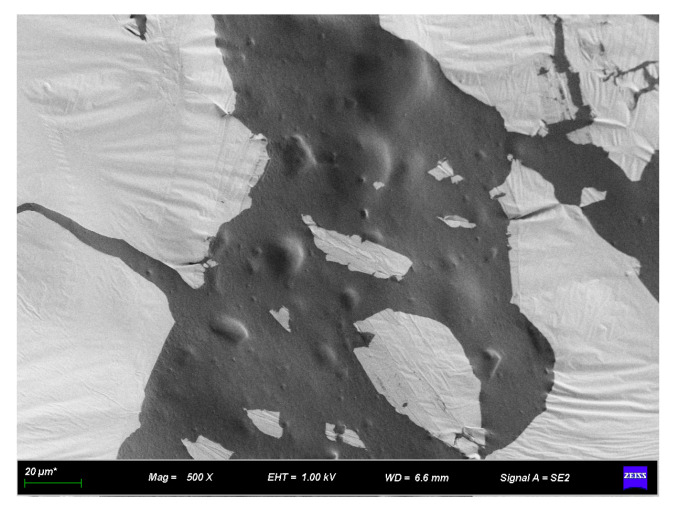
SEM appearance of rubber–diatomite asphalt.

**Figure 12:**
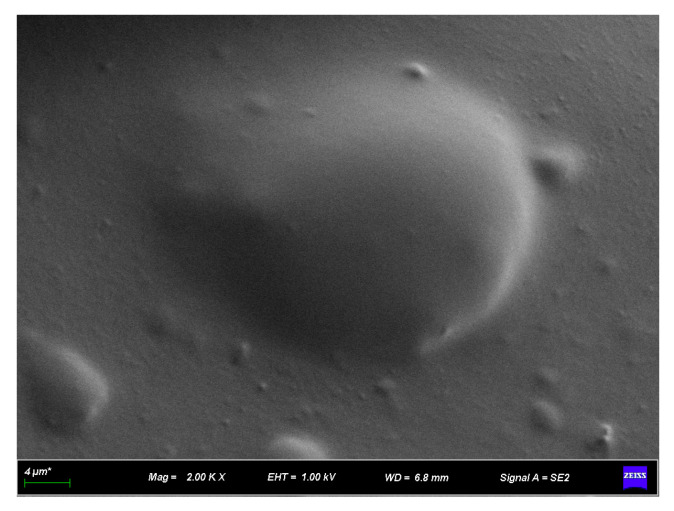
SEM appearance of rubber–diatomite asphalt.

Figure 9, Figure 10, Figure 11 and Figure 12 present SEM images of base asphalt, rubber asphalt, and rubber–diatomite composite-modified asphalt. In Figure 9, the SEM image of the base asphalt appears relatively smooth and without visible wrinkles on the surface. However, with the addition of rubber powder, the surface begins to exhibit roughness and signs of high-temperature shearing are evident. The rubber powder particles show distinct angular characteristics that indicate the swelling reaction between the rubber powder and the asphalt. Traditional rubber asphalt is primarily characterized by the swelling mechanism of oil absorption and network filling. Following shearing at high temperatures, the rubber powder particles experience varying degrees of volume expansion within the asphalt.

With the inclusion of diatomite, the protrusions and wrinkles of the surface become more prominent. These observations indicate that asphalt, rubber, and diatomite are closely intertwined and asphalt includes both. Examination of the interface of individual diatomite and asphalt shows that the modification of asphalt by diatomite does not involve a chemical reaction. Instead, it primarily constitutes a process of physical absorption. At high temperatures, the asphalt penetrates the internal cavities of the diatomite and creates interlocking and anchoring effects, resulting in a strong mechanical bond. Such an interplay leads to a substantial performance enhancement of composite-modified asphalt.

**Figure 13:**
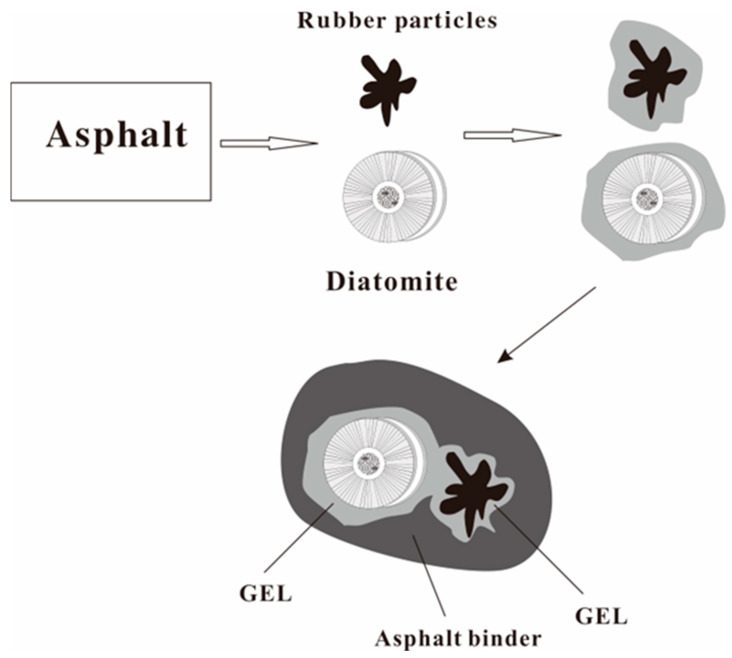
Model illustration of rubber–diatomaceous earth-modified asphalt process.

Figure 13 presents a process model of an asphalt-modified rubber–diatomite composite that illustrates the multiple roles that diatomaceous earth and rubber play in the modification process:

(1) Rubber and diatomite exhibit the following functions. At high temperatures, they physically adsorb easily permeable and diffusible components. As the temperature decreases, these components coalesce and harden in the internal cavities and surrounding areas, generating an anchoring and stable effect in the asphalt. As a result, this leads to an increase in the resistance to asphalt aging.

(2) Diatomite contributes to improving the adhesion and mechanical locking of modified asphalt. At the same time, it reduces high-temperature fluidity, increases viscosity, and enhances thermal stability.

(3) It is noteworthy that rubber and diatomite do not undergo chemical reactions with asphalt. Their interaction primarily involves a process of physical blending. Excessive amounts of any of the components may lead to a reduction in the physical properties of asphalt.

## 4. Performance Evaluation of Modified Asphalt Mixtures 

### 4.1. The Marshall Test of Asphalt Mixtures

Rubber-modified asphalt, rubber–SBS-modified asphalt, and a rubber–diatomite-modified asphalt mixture were tested in the optimized rubber–diatomite-modified asphalt test. According to the Marshall test in “Technical Specifications for Construction of Highway Asphalt Pavements” (JTGF40-2004), gross bulk density, stability, flow rate, porosity ratio, mineral clearance ratio, and asphalt saturation index were measured, and finally, the optimal amount of asphalt was determined. The optimal asphalt contents of rubber asphalt mixture, rubber–SBS-modified asphalt mixture, and rubber–diatomite-modified asphalt mixture were 5.6%, 5.8%, and 6.0%, respectively. The results of the Marshall test of the asphalt mixture are presented in Table 10. Compared with rubber-modified asphalt, the Marshall stability of rubber–diatomite asphalt exhibits an increase of 2.83 KN, indicating that the addition of diatomite can enhance the stability of the asphalt mixture.

### 4.2. The High-Temperature Stability of Asphalt Mixture

#### 4.2.1. The Wheel Tracking Test of Asphalt Mixture 

Wheel tracking tests were carried out on different asphalt mixtures and rutted deformation and dynamic stability were recorded for 45 min and 60 min. The results are shown in Table 11. Table 11 shows that the dynamic stability of rubber–diatomite asphalt increased by 1035 (times/mm) and 410 (times/mm) compared to rubber-modified asphalt and rubber-modified asphalt with SBS, and the deformation of rubber–diatomite composite-modified asphalt is also the smallest among the three types of asphalt.

The results showed that diatomite is capable of improving wheel tracking performance and noticeably reducing rut deformation. This is chiefly ascribed to the fact that rubber and diatomite play a cross-linking role in the asphalt modification process and form a new stable structure with asphalt. Therefore, under the repeated action of wheel load, the skeleton structure of the mixture is strengthened, and the aggregate does not produce large displacements, thus improving the rutting resistance of the rubber–diatomite composite-modified asphalt mixture [42]. 

#### 4.2.2. The Wheel Tracking Test of Asphalt Mixture Aging 

In order to investigate the impact of high-temperature aging properties of asphalt mixture in the presence of complex conditions, short-term aging and long-term aging tests were performed on the asphalt mixture of rubber asphalt, rubber–SBS-modified asphalt, and rubber–diatomite-modified asphalt. Short-term aging: After homogenization at the specified mixing temperature, the loose mixture was heated in a forced air oven at 135 ± 1 °C for 4 h ± 5 min. Long-term aging: After short-term aging, the molded samples were heated in a forced-air oven at a temperature of (85 ± 1) °C for 120 h [43]. The test specimens were then heated in a forced-air oven at (85 ± 1) °C for 4 h ± 5 min. The dynamic stability test of rutting has been provided in Table 12.

After the thermal oxidative aging test, the dynamic stability of different asphalt mixtures increased. From the comparison investigation, it can be seen that regardless of what type of modifier is used, the dynamic stability after aging is substantially improved compared to before aging, and this increase is mainly concentrated in the short-term aging stage. However, the increased degrees of dynamic stability of rubber asphalt, rubber–SBS-modified asphalt, and rubber–diatomite-modified asphalt mixture are different. The ratios of dynamic stability of RDS_1_ and RDS_2_ were employed to evaluate the effect of the asphalt mixture with thermal oxygen aging on high-temperature stability. RDS_1_ and RDS_2_ are calculated according to Equations (5) and (6), respectively:

RDS_1_ = DS_2_/DS_1_ (5)

RDS_2_ = DS_3_/DS_1_ (6)

where RDS_1_ and RDS_2_ represent the dynamic stability ratios of the asphalt mixture after the thermal oxidative aging test, DS_1_ is the dynamic stability of the asphalt mixture before thermal oxidative aging, and DS_2_ and DS_3_ are, respectively, the dynamic stability of the asphalt mixture after short-term and long-term thermal oxidative aging.

The order of RDS1 is as follows: 

rubber asphalt mixture < rubber–SBS-modified asphalt mixture;

rubber–SBS-modified asphalt mixture < rubber–diatomite-modified asphalt mixture. 

The order of RDS_2_ is as follows: 

rubber asphalt mixture < rubber–SBS-modified asphalt mixture;

rubber–SBS-modified asphalt mixture < rubber–diatomite-modified asphalt mixture.

This indicates that both SBS and diatomite are capable of improving the thermal oxidative aging resistance of the asphalt mixture, and the rubber–diatomite extract-modified asphalt mixture exhibits the best thermal oxidative aging resistance, which is consistent with the evaluation results of high-temperature stability. This is essentially attributed to the fact that diatomite is able to configure a unique embedded structure with the asphalt and lock the asphalt, which acts as a barrier against oxygen and extends the volatilization path of the light components in the asphalt. This fact inhibits the oxidation reaction of asphalt and the volatilization of the light components, thus reducing the rate of thermal oxidative aging of asphalt and improving the resistance to thermal oxidative aging of the asphalt mixture.

### 4.3. The Long-Term Water Stability Performance Test

#### 4.3.1. The Cyclic Freeze–Thaw Splitting Test

**Figure 14:**
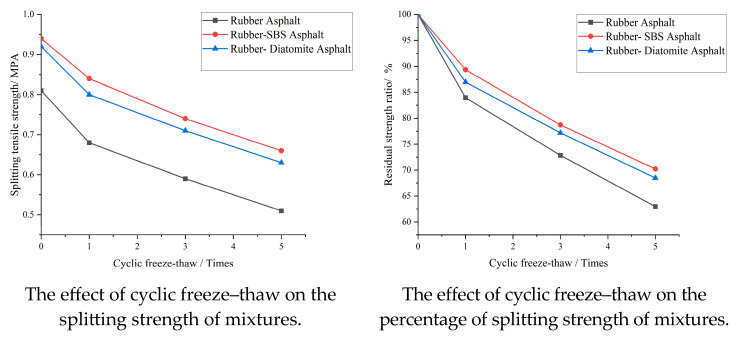
Results of cyclic freeze–thaw splitting test.

Considering the actual pavement environment, in order to more accurately evaluate the long-term water stability performance of various types of asphalt mixtures, one, three, and five freeze–thaw cycles were added to the main freeze–thaw splitting test in the cyclic freeze–thaw test to simulate the long-term use of the mixture in the actual environment. The samples filled with vacuum water were frozen in a constant-temperature refrigerator at −18 °C for 16 h and then placed in a constant-temperature water bath at 60 °C for 24 h, which was regarded as a completion of a freeze–thaw cycle. The results of the freeze–thaw splitting test are presented in Table 13 and Figure 14.

After three cycles of freeze–thaw tests, the splitting strength ratio of various modified asphalt mixtures decreased to a certain extent, and after five freeze–thaw test cycles, the three types of asphalt mixtures decreased significantly. The splitting strength curves from high to low were SBS rubber particles, rubber–diatomite particles, and rubber particle-modified asphalt mixtures. This reveals that diatomite is able to provide the mixture of rubber particles with a higher splitting strength in the freeze–thaw cycle, but the improvement effect is less than that of the SBS-based modifier.

The obtained results indicate that the addition of diatomite and rubber particles reduces the freeze–thaw splitting strength of asphalt mixtures. This also has some impact on the water stability of the asphalt mixture, which may be due to the rubber and diatomite particles added to the asphalt mixture as elastomers. After milling and molding, the particles in the asphalt mixture will be in an extruded state with some internal stress. This internal stress along with the expansion force caused by water freezing leads to an increase in the damage to the asphalt mixture and thus reduces the stability of the asphalt mixture in water. When SBS modification is employed, the negative effect of rubber particles on water stability is relatively weak. The SBS-modified asphalt mixtures exhibit strong bonding properties, which are capable of limiting the damage caused by the internal stress of rubber particles and the freezing and expansion forces of water, thus reducing the amount of asphalt mixture damage.

#### 4.3.2. The Cyclic Freeze–Thaw Drying Aging Splitting Test

**Figure 15:**
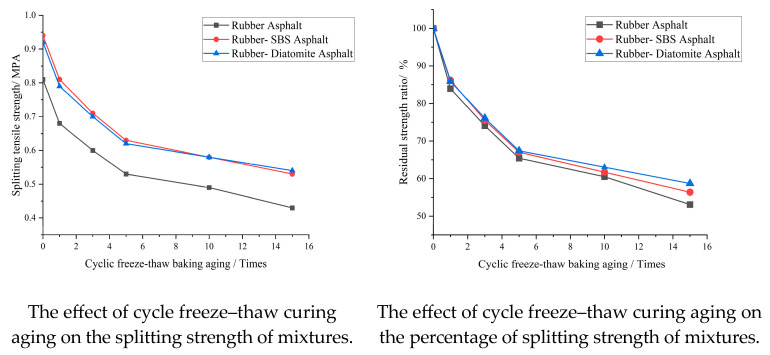
Results of cyclic freeze–thaw curing aging splitting test.

To simulate the aging process of the asphalt mixture at natural high temperatures, “drying” conditions are added based on the freeze–thaw split test. It is commonly employed to evaluate the long-term water stability performance of different asphalt mixtures under freezing, thawing, and curing (drying) conditions to further comprehensively evaluate the advantages and disadvantages of treatment approaches. After the samples filled with vacuum water were frozen for 16 h in a refrigerator with a constant temperature of −18 °C, they were placed in a water bath with a constant temperature of 60 °C for 24 h, and then the asphalt mixture samples were placed at a temperature of 60 °C for 12 h. After taking out the samples, they were placed in a water tank with a constant temperature of 25 ± 0.5 °C for 2 h, which was considered the completion of the freeze–thaw drying cycle. After the fifth cycle, the mixture was immersed in a constant-temperature water tank at 25 °C for 2 h, and the splitting strength of the mixture could be determined according to the requirements of the freeze–thaw split test [44]. Based on Ma’s research, we selected 1, 3, 5, 10 and 15 cycles for testing [45]. After repeating the cycle 1, 3, 5, 10, and 15 times, the ratio of splitting strength of freezing, thawing, and drying and splitting strength of freezing, thawing, and drying was obtained by splitting test. The results of the freeze–thaw splitting test are presented in Table 14 and Figure 15.

After adding the mixture to curing, the splitting strength of each asphalt mixture decreased more obviously, and the fastest decreasing strength was observed for the rubber–asphalt mixture. The decreasing trend of rubber–SBS-modified asphalt and rubber–diatomite-modified asphalt was similar. After 15 cycles of freezing, thawing, and drying, the residual strength ratio of the rubber–diatomite mixture was reported to be the highest, and the excellent rubber–SBS-modified asphalt and rubber asphalt are the best. The addition of diatomite makes the performance stability of the mixture superior to that of rubber–SBS-modified asphalt in a complex environment, indicating that the modified diatomite enhances the water stability performance and high-temperature stability performance of the asphalt mixture. This is essentially ascribed to the fact that the diatomite makes the asphalt mixture more compact, thus enhancing the damage resistance of the asphalt mixture.

## 5. Conclusions and Further Research

### 5.1. Conclusions

The preparation of rubber–diatomite-modified asphalt was methodically examined by employing the surface response methodology. Different performance tests, microscopic analysis, and road performance verification were carried out on the appropriately prepared modified asphalt and the following results can be deduced:

(1) There exists a nonlinear functional relationship between the rubber and diatomite content of treated soil and asphalt properties. According to the surface response method, in rubber–diatomite-modified asphalt, the reasonable content of rubber is 22% and the content of diatomite is 4.0%.

(2) According to the results of scanning electron microscopy and infrared spectrum test, by adding rubber and diatomite, diatomite incorporates into the modifying process of physical mixing and adsorption of asphalt, which can be combined with rubber and asphalt to configure a stable composite structure.

(3) The optimal degree of rubber–diatomite-modified asphalt AC-13 is obtained as 6.0%. Compared to the rubber-modified asphalt mixture, the rubber–diatomite mixture has less wheel tracking test deformation and correspondingly higher stability. And it has about the same properties as rubber–SBS-modified asphalt.

(4) The addition of rubber and diatomite particles can improve the high-temperature stability and low-temperature cracking resistance of asphalt mixtures, but the improvement effect is weaker than that of SBS. This is because rubber and diatomite particles are mainly used for physical combination to strengthen asphalt mixtures, and their effect is weaker than the chemical modification of SBS. However, after the cyclic curing freeze–thaw test, the rubber–diatomite composite-modified asphalt mixture and rubber–SBS-modified asphalt mixture maintain similar water stability properties, indicating that the addition of diatomite can improve the performance of asphalt mixtures in complex material environments.

### 5.2. Further Research

Through the above research results, the application prospects and future research directions of rubber–diatomite-modified asphalt are summed up below.

(1) The use of waste rubber powder and diatomite-modified asphalt has environmental greenness potential, which is of great significance in the construction of highways and bridges in sustainable development and a green environment.

(2) The road performance of rubber–diatomite-modified asphalt has been improved, and it is suitable for use in high-temperature areas and applicable to heavy-traffic pavement.

(3) We have broadened the application of rubber–diatomite-modified asphalt in grading. More in-depth research should determine the best amount of asphalt, as well as the cost savings of this mixture for best road performance.

(4) We should vigorously promote the application of rubber–diatomite-modified asphalt in the actual project, to examine its actual performance and to accumulate more practical experience in engineering applications.

## Figures and Tables

**Table 1:** Technical indexes of 70# asphalt.

Technical Parameter		Unit	Requirements	Test Value
Penetration (25 °C, 5 s, 100 g)		mm	60~80	66
Softening Point		°C	≥46	47.2
15 °C Ductility		cm	>100	>100
After RTFOT	Mass Variation	%	±0.8	−0.2
	Residual penetration ratio	%	≥61	67.5
	Ductility	cm	≥6	7.1

**Table 2:** Technical indicators of rubber powder.

Technical Parameters		Unit	Requirements	Test Value
Physical properties	Relative density	-	1.23	1.23
	Humidity	%	0.2	0.2
	Metal content	%	0.011	0.011
	Fiber content	%	0.078	0.078
Chemical properties	Ash content	%	6.12	6.12
	Acetone extract	%	4.03	4.03
	Carbon black content	%	31.244	31.244
	Rubber hydrocarbon content	%	55.46	55.46

**Table 3:** Technical indicators of diatomite.

Technical Parameters	Unit	Test Value
Density	g/cm^3^	2.15
Average particle size	μm	15.22
Oil absorption rate	g/cm^3^	1.45
Specific surface area	g/cm^2^	47.65
Void volume	g/cm^3^	0.02
Average radius	nm	2.12

**Table 4:** Main performance indicators of aggregates.

Indexes		Test Value	Requirements
Apparent relative density (g/cm^3^)	gravel (10–20 mm)	2.73	≥2.60
	gravel (5–10 mm)	2.68	
	grit (0–5 mm)	2.75	≥2.50
Crushing value (%)		17	≤26.00
Los Angeles wear loss (%)		25	≤28.00
Sand equivalent (%)		75	≥60.00

**Table 5:** Quality testing results of mineral powder.

Indexes		Unit	Requirements	Test Result	Test Method
Apparent density, not less than		t/m^3^	2.50	2.714	T 0352-2000
Moisture content, not greater than		%	1	0.18	T 0103
Particle size range	<0.6 mm	%	100	100.0	T 0351-2000
	<0.15 mm	%	90~100	94.8	
	<0.075 mm	%	75~100	86.7	

**Table 6:** Test schemes of modified asphalt.

Serial Number	Asphalt (%)	Rubber Powder Proportion (%)	Diatomite Proportion (%)
A1	100	16	4
A2	100	16	8
A3	100	16	12
A4	100	20	4
A5	100	20	8
A6	100	20	12
A7	100	24	4
A8	100	24	8
A9	100	24	12

**Table 7:** Design of gradation.

Sieve Size (mm)	16	13.2	9.5	4.75	2.36	1.18	0.6	0.3	0.15	0.075
Upper limit	100	100	70	38	28	24	18	14	11	7
Lower limit	100	90	50	20	15	12	8	5	3	2
Synthetic grade	100	99.7	64.3	31.0	22.7	16.8	11.6	8.9	6.9	5.5

**Table 8:** Test results of rubber–diatomite compound-modified asphalt index.

No.	Asphalt Type	Penetration (0.1 mm)	Softening Point (°C)	Ductility (cm)	Rotational Viscosity (Pa·s)
A1	Rubber–Diatomite 16 + 4	40.2	63.9	6.9	2.31
A2	Rubber–Diatomite 16 + 8	39.4	64.6	5.2	2.39
A3	Rubber–Diatomite 16 + 12	38.89	66.3	4.6	2.49
A4	Rubber–Diatomite 20 + 4	38.78	68.8	8.6	2.31
A5	Rubber–Diatomite 20 + 8	37.77	69.2	7.6	2.54
A6	Rubber–Diatomite 20 + 12	36.93	69.4	6.8	2.87
A7	Rubber–Diatomite 24 + 4	37.27	71.5	7.0	2.48
A8	Rubber–Diatomite 24 + 8	33.57	76	5.8	2.77
A9	Rubber–Diatomite 24 + 12	32.45	78	4.9	3.21

**Table 9:** Optimization of the experimental plan and validation of the asphalt performance.

Test	Asphalt (%)	Rubber (%)	Diatomite (%)	Penetration (25 °C, 0.1 mm)	Softening Point (°C)	Ductility (5 °C/cm)	Rotational Viscosity (Pa·s)
Predicted value	100	22	4	38.2	69.7	8.36	2.389
Experimental value	100	22	4	38.1	70.5	8.3	2.391

**Table 10:** Asphalt Marshall test results.

Types of Asphalt	Optimum Amount of Asphalt (g/cm^3^)	Density (g/cm^3^)	VV (%)	VFA (%)	VMA (%)	MS (KN)	FL (mm)
Rubber asphalt	5.6	2.57	4.4	73.8	16.8	10.60	3.21
Rubber–SBS asphalt	5.8	2.69	4.5	73.9	17.2	13.31	3.20
Rubber–Diatomite asphalt	6.0	2.70	4.4	73.7	17.1	13.44	3.14

VV: void rate of asphalt mixture specimens; VMA: mineral clearance rate of asphalt mixture specimens; VFA: effective asphalt saturation of asphalt mixture specimens; MS: asphalt Marshall stability; FL: asphalt flow value.

**Table 11:** Asphalt wheel tracking test results.

Types of Asphalt	Time1 (min)	Time2 (min)	*D*_1_ (mm)	*D*_2_ (mm)	Dynamic Stability (times/mm)
Rubber asphalt	45	60	2.669	2.860	3298
Rubber–SBS asphalt	45	60	2.440	2.605	3818
Rubber–diatomite asphalt	45	60	2.381	2.530	4228

**Table 12:** Asphalt aging rut test results.

Types of Asphalt	Dynamic Stability/(times-mm-1)		
	Unaging	Short-Term Aging	Long-Term Aging
Rubber asphalt	3298	4452	4979
Rubber–SBS asphalt	3818	4848	5406
Rubber–diatomite asphalt	4228	5158	5538

**Table 13:** Results of cyclic freeze–thaw splitting test.

Types of Asphalt	Number of Freeze–Thaw Cycles	Split Strength (MPA)	Residual Strength Percentage (%)
Rubber asphalt	0	0.81	100.00
	1	0.68	83.95
	3	0.59	72.84
	5	0.51	62.96
Rubber–SBS asphalt	0	0.94	100.00
	1	0.84	89.36
	3	0.74	78.70
	5	0.66	70.21
Rubber–diatomite asphalt	0	0.92	100.00
	1	0.8	86.96
	3	0.71	77.17
	5	0.63	68.48

**Table 14:** Results of cyclic freeze–thaw curing aging test.

Types of Asphalt	Number of Freeze–Thaw Cycles Drying Aging	Split Strength (MPA)	Residual Strength Percentage (%)
Rubber–SBS asphalt	0	0.81	100.00
	1	0.68	83.95
	3	0.6	74.07
	5	0.53	65.43
	10	0.49	60.49
	15	0.43	53.09
Rubber–SBS asphalt	0	0.94	100.00
	1	0.81	86.17
	3	0.71	75.53
	5	0.63	67.02
	10	0.58	61.70
	15	0.53	56.38
Rubber–diatomite asphalt	0	0.92	100.00
	1	0.79	85.87
	3	0.70	76.09
	5	0.62	67.39
	10	0.58	63.04
	15	0.54	58.70

## Data Availability

The data used to support the findings of this study are included within the article.
